# Chromosome Y Centromere Array Deletion Leads to Impaired Centromere Function

**DOI:** 10.1371/journal.pone.0086875

**Published:** 2014-01-22

**Authors:** Alison N. Graham, Paul Kalitsis

**Affiliations:** 1 Murdoch Childrens Research Institute, Melbourne, Victoria, Australia; 2 Department of Paediatrics, University of Melbourne, Melbourne, Victoria, Australia; Leibniz-Institute of Plant Genetics and Crop Plant Research (IPK), Germany

## Abstract

The centromere is an essential chromosomal structure that is required for the faithful distribution of replicated chromosomes to daughter cells. Defects in the centromere can compromise the stability of chromosomes resulting in segregation errors. We have characterised the centromeric structure of the spontaneous mutant mouse strain, BALB/cWt, which exhibits a high rate of Y chromosome instability. The Y centromere DNA array shows a de novo interstitial deletion and a reduction in the level of the foundation centromere protein, CENP-A, when compared to the non-deleted centromere array in the progenitor strain. These results suggest there is a lower threshold limit of centromere size that ensures full kinetochore function during cell division.

## Introduction

The centromere is a multi-subunit DNA/protein structure that assembles a mature kinetochore after DNA replication in order to efficiently capture spindle microtubules for chromosome segregation. In mammals, the underlying DNA is mostly composed of AT-rich satellite DNA spanning a range of 90 kb to several megabases [1], [2]. A subset of this repetitive DNA is functionally marked by centromere-specific nucleosomes containing the essential histone H3 variant, CENP-A plus other constitutive centromere proteins [3], [4]. This chromatin is the foundation layer onto which mitotic-specific proteins assemble to form a mature kinetochore that modulates the timing and fidelity of chromosome attachment and movement during mitosis and meiosis [5], [6].

In the mouse, the functional centromere of the autosomes and the X chromosome is localised to the 120 bp minor satellite repeat DNA, which is organised in a head-to-tail arrangement spanning 300–600 kb [7], [8]. In contrast, the Y chromosome centromere is relatively small, containing only 90 kb of the diverged minor satellite repeat DNA known as Ymin [1]. The Y chromosome is an excellent model chromosome for studies into structural and functional centromere biology because it is haploid and has a diverged, small centromere.

To investigate the role of the Y centromere structure on chromosome stability we have used two spontaneous laboratory mouse mutant strains, BALB/cWt and A/HeJ, that are associated with a high incidence of hermaphroditism [9], [10]. Both mutant strains display high rates of Y chromosome non-disjunction when compared to inbred strains that carry their progenitor Y chromosome [11], [12], [13]. It has been hypothesized that mutations at or near the Y centromere may contribute to the chromosome instability phenotype [14]. This is true for centromere DNA mutations in the single cell model organism *Saccharomyces cerevisiae*, which can compromise the fidelity of chromosome segregation [15], [16], [17], [18]. In this study we make use of the complete mouse Y centromere sequence map to test whether centromere DNA mutations are responsible for the chromosome instability phenotype of the Wt Y chromosome.

## Materials and Methods

### Mouse Strains

The BALB/cWtEiJ and BALB/c mouse strains were purchased from the Jackson Laboratory, USA and the Walter and Eliza Hall Institute, Australia, respectively. Research involving mice was conducted according to national guidelines, and was approved by the Murdoch Childrens Research Institute Animal Ethics Committee, Project No. A711.

### Cell Culture

Primary mouse embryonic fibroblasts (PMEFs) were derived according to Mann [19], [20] at 13.5 d post coitum (dpc) and grown until confluency. Cells were then frozen and cryogenically preserved. Upon thawing, one vial of cells was divided in to two 10-cm plates with DMEM (Merck Millipore) supplemented with 12% FBS (Bovogen), non-essential amino acids (Sigma-Aldrich), penicillin and streptomycin (Sigma-Aldrich), 0.1 mM β-mercaptoethanol and L-glutamine.

### PFGE and Southern Blot Hybridisation

For long range DNA separation, genomic DNA was prepared using standard methods. Agarose-embedded DNA was digested overnight with 40 units of the selected restriction endonuclease. Digested DNA was separated using a Bio-Rad Chef Mapper; run conditions are described in the figure legend. Agarose gels were blotted onto Hybond-N+ (GE Healthcare) using standard methods. DNA probes were labelled with Ready-To-Go DNA Labeling Beads (GE Healthcare) incorporating dCTP alpha^32^P (Perkin Elmer). Blots were hybridised in 5 ml of Church buffer at 67°C overnight and then washed in 1×SSC/0.1% SDS at 68°C.

### Immuno-FISH

PMEF cells were treated with the addition of 0.1 µg/ml demecolcine (Sigma-Aldrich) to the media for three hrs before cells were trypsinized, washed in PBS and resuspended at a concentration of 1.5×10^5^ cells/ml in 75 mM KCl hypotonic solution for 10 min. 200 µl of cells were cytospun on to glass slides and immunofluorescence was carried out as described (Kalitsis et al 2003) using a monoclonal rabbit anti-mouse Centromere protein-A (CpA) (C51A7; Cell Signaling) primary antibody and donkey anti-rabbit IgG Alexa Fluor 488 (Molecular probes) secondary antibody. CENP-A levels were quantified as described in *Imaging and Quantification*.

FISH probes were directly labeled with Alexa Fluor dyes using a FISH Tag DNA labeling kit (Invitrogen) in accordance with the manufacturer’s instructions. The following FISH probes were used to identify X and Y-chromosomes after CENP-A quantification, Mgclh pericentromere and Ymin centromere, respectively. The tandemly repeated Mgclh gene was amplified with the following primers; Mgclh-Bam 5′-TATGGATCCTGAAGGCCTATTTTCCATTTCC-3′ and Mgclh-SacII 5′-AATCCGCGGCAGGCAACACACATGTGGAC-3′ and then the 2.4 kb fragment was cloned into pBluescriptIISK+ vector (Stratagene). FISH was performed as described in [1].

### Imaging and Quantification

For CENP-A quantification, 26 metaphase spread z-stack images were captured at 0.2-µm intervals to span the depth of the mitotic cell using an Axioimager.M1 microscope (Zeiss)/AxioCam MRm CCD camera and constrained iterative deconvolution processed using AxioVision (AxioVs40v4.6.1.0) software (Zeiss). The z stack was used to generate a maximum intensity projection image and analysis was analysed as previously described in [21]. Fluorescence in situ hybridisation (FISH) images of the previously imaged metaphase spreads were used to identify the X and Y chromosome in each metaphase spread. The mean intensity of the CENP-A signal was measured within a 0.2 µm diameter circular area around each of the Y chromosome sister centromere signals (Y_cen1_ and Y_cen2_) and also of the X chromosome centromeres (X_cen1_ and X_cen2_). To account for background signal, four randomly selected non-centromeric regions from the Y (Y_b1–b4_) and X (X_b1–b4_) chromosomes were measured for mean signal intensity using a 0.2 µm diameter circular area. The mean signal intensity was averaged and subtracted from the respective centromeric CENP-A signal. Background-subtracted CENP-A levels were then expressed as a ratio of Y^CENP-A^/X^CENP-A^ for each metaphase cell.

## Results

### Interstitial Deletion of the Y Centromere DNA Array

Bean and colleagues have postulated that the Wt Y chromosome non disjunction was due to a ‘sub-optimal’ centromere [14]. One suggested possibility for this is that the underlying centromere DNA has undergone a *de novo* mutation. To test this hypothesis we probed the centromeric region with the Y-specific centromere sequence, Ymin. This sequence has been shown to bind to the foundation centromere protein, CENP-A [1].

**Figure 1:**
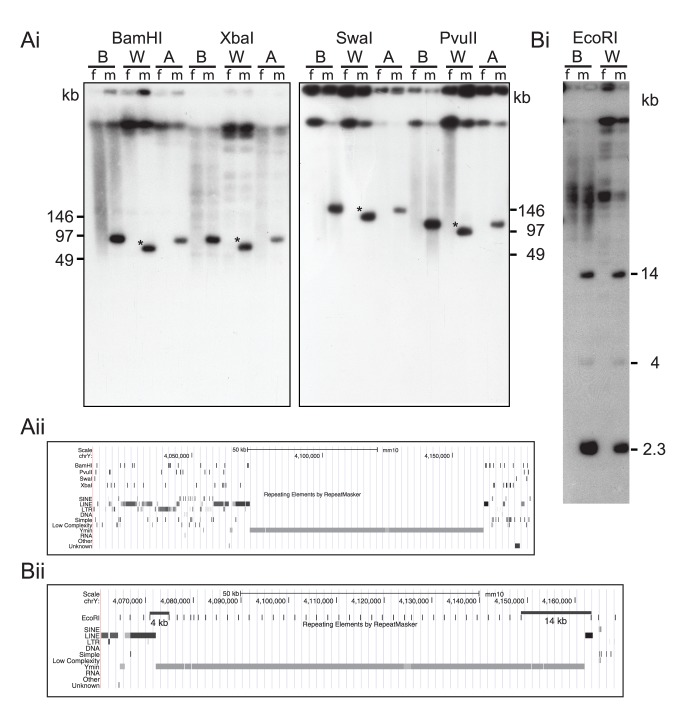
Long range centromere DNA analysis of BALB/c, BALB/cWt and A/HeJ strains. (Ai) DNAs from male-female pairs of each strain were digested with four restriction endonucleases that cut outside of the Ymin array. The Y chromosome unstable BALB/cWt strain shows a consistent decrease in length, asterisk band, when compared to the progenitor BALB/c strain. (Aii) Predicted restriction map of enzymes that flank the Ymin array. (Bi) Internal organisation of the Ymin array in the BALB/c and BALB/cWt mouse strains. The HOR unit of 2.3 kb is release with *Eco*RI, together with p and q junction fragments of 4 and 14 kb, respectively. (Bii) UCSC Genome Browser snapshot of the *Eco*RI sites within and around the Ymin region. PFGE run conditions, 0.5×TBE, 6 V/cm, 2–40 sec pulse switch time, 18 hr, 0.9% agarose.

Long-range PFGE mapping was performed on BALB/c (the progenitor to strain WtEiJ), BALB/cWtEiJ and AHe/J (another chromosome Y unstable strain). DNA from male and female pairs were digested with four restriction enzymes that flank the whole Ymin array. The expected fragment sizes predicted from the C57BL/6J reference genome were as follows: *Bam*HI (90.8 kb), *Xba*I (96.9 kb), *Swa*I (162 kb), *Pvu*II (115 kb). BALB/c and AHe/J male animals showed the expected sizes for each enzyme. In contrast, the BALB/cWtEiJ male strain showed a consistent 18 kb reduction in length for all four flanking enzymes (Fig. 1). Since the Ymin array spans a distance of 90 kb, the Wt Y centromeric array shows a reduction of approximately 20% when compared with control Y centromeres from the *M. m. molossinus* sub species.

In order to determine where the deletion had occurred within the Y centromere array we digested genomic DNA with *Eco*RI. This digestion releases the high order repeat (HOR) array unit of 2.3 kb plus the flanking p and q junction fragments of 4 and 14 kb, respectively (Fig. 1). Both expected p and q bands appeared as predicted from the reference genome. The higher order repeat band was also present but at a slightly reduced intensity when compared with the ancestral BALB/c Ymin array. No additional bands were present in the Southern blot. This mapping analysis suggests that the most likely region of the deletion is within the high order repeat array.

### CENP-A Reduction at the Wt Y Kinetochore

The above centromeric DNA analysis showed that a deletion within the Ymin array correlated with the Wt Y unstable chromosome. This DNA analysis alone cannot confidently predict the size of the kinetochore because centromeres are not always DNA sequence dependent. Examples of this are shown in the appearance of neocentromeres throughout genome evolution and in humans with chromosomal rearrangements. The existence of neocentromeres provides evidence for an epigenetic component to centromere establishment and maintenance [22]. The primary candidate for this mark is the CENP-A protein, upon which most kinetochore proteins are dependent [3], [23]. To ascertain the size of the kinetochore within the Wt Y centromere, we measured the amount of CENP-A compared with the progenitor BALB/c Y centromere using a mouse-specific CENP-A antibody.

At least 16 metaphase spreads from four 13.5 day old embryos for each BALB/c and Wt strain were measured for amounts of the CENP-A protein at sister centromere pairs. The Y^Cenpa^ levels were normalised against the single X centromere pair in order to correct for variation in fluorescence between metaphase cells. The Y^Cenpa^/X^Cenpa^ ratios for the progenitor BALB/c strain showed a ratio of 0.92 suggesting that the amount of CENP-A at the Y centromere is only marginally less than that at the X centromere. This is despite the length of the Y centromere DNA being only 90 kb, compared with hundreds of kilo base pairs for the minor satellite-containing centromeres [1], [7], [8]. It has been shown previously that the kinetochore forms within a subset of the larger minor satellite arrays [8], [24], while the kinetochore occupies the majority of the Y centromere array [1].

**Figure 2:**
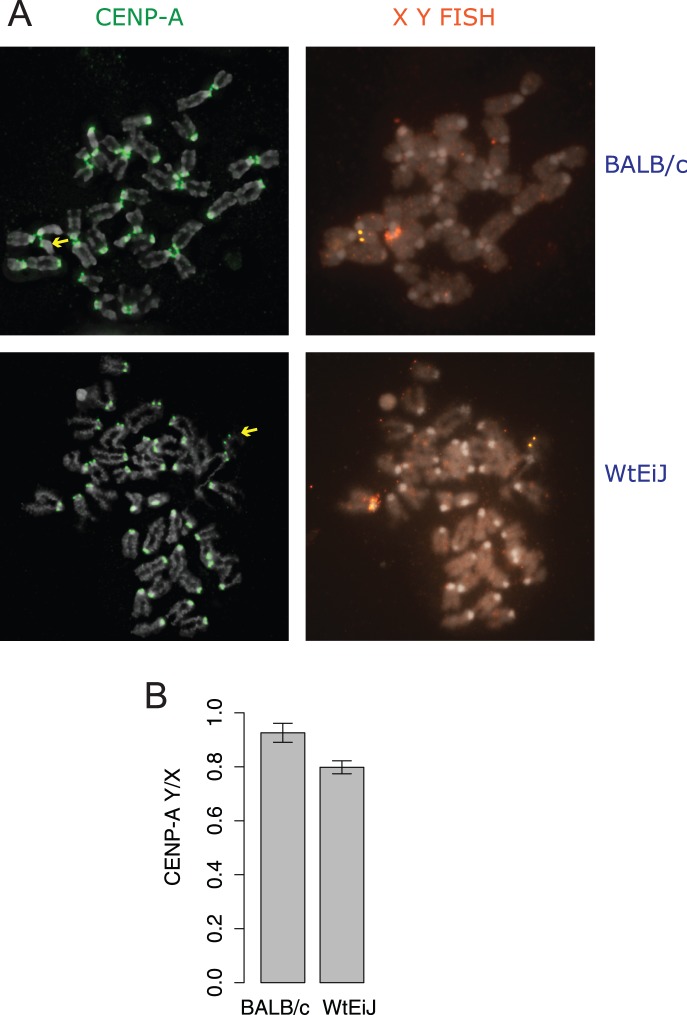
Quantification of CENP-A at chromosome X and Y centromeres. (A) Metaphase chromosomes from representative BALB/c and BALB/cWt cells showing CENP-A signals as (green) paired dots for each chromosome. FISH probes, Mgclh (red) and Ymin (yellow) were used to identify the X and Y chromosomes, respectively. (B) Y CENP-A levels were normalised against the X centromere and shown as a ratio of CENP-A^Y/X^. *P* = 0.029, student’s t test, error bars represent the standard error of the mean, n = 70 cells.

When the averaged levels of CENP-A on the BALB/c Y chromosome were compared with the Wt Y chromosome, we observed a significant downward shift in Y^Cenpa^/X^Cenpa^ ratios from 0.92 to 0.80 (Fig. 2B). This represents a 13% drop in CENP-A levels on the Wt Y centromere. This reduction in CENP-A at the Wt Y centromere is seen in association with the interstitial deletion of the Ymin array as was observed using PFGE analysis.

## Discussion

**Figure 3:**
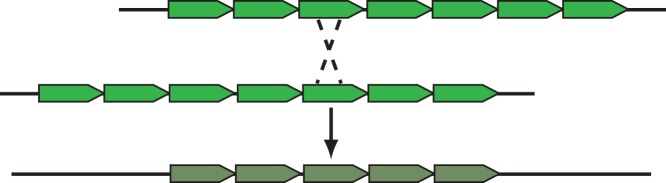
Model for interstitial deletion of Ymin satellite array. An out-of-register cross over event between Ymin higher order repeat units on sister chromatids produces a shortened centromere array.

We have previously shown that the mouse Y centromere DNA array is remarkably stable in the short term, with no detectable change in array length spanning over 700 generations in five separate inbred mouse strains that carry the *M.m. molossinus* Y chromosome [1]. Each of these strains contains the same HOR unit structure and the same overall Ymin array length of 90 kb. In contrast, the Wt Y centromere shows an interstitial deletion of 18 kb, which has occurred *de novo* in this strain. This represents a 20% decrease in the amount of Ymin DNA at the Wt Y centromere. Restriction mapping analysis of the p and q Ymin junction regions showed that the expected flanking fragments remained intact and suggests that the deletion has occurred within the Ymin HOR array. This molecular analysis of the Wt Y centromere supports previous models of satellite centromere DNA expansion and contraction via an out-of-register chromatid exchange event between homologous HOR units (Fig. 3). This deletion event has most likely occurred during either meiosis or germ cell development.

If the interstitial deletion of the Ymin array is the cause of the Wt Y centromere impairment, then why doesn’t CENP-A just spread to adjacent regions to compensate? Human neocentromeres and evolutionary new centromeres are atypical centromeres that show that CENP-A can seed into new satellite-free DNA regions, usually triggered by a chromosomal rearrangement [22]. In contrast, the reduced Wt Y CENP-A signal still coincides with the Ymin array and shows no evidence of expansion into bordering regions. Furthermore, the Wt Y centromere boundary edges remain intact as shown by the Ymin junction restriction site analyses (Fig. 1B). Therefore, it appears that CENP-A is restricted from spreading by pre-existing unknown boundary elements.

A reduction/deletion in the underlying centromere DNA might be expected to produce a decrease in the size of the kinetochore because of its known repeating subunit organization, assuming there is no spreading of the CENP-A and that the deleted DNA spans the CENP-A region [25], [26], [27]. Our CENP-A quantification analysis has shown a concomitant reduction when compared with the progenitor BALB/c centromere. This decrease in CENP-A size appears to have a subtle effect on chromosome segregation as the mitotic defect is only observed in the first few cell divisions of the BALB/cWtEiJ embryo [14].

One of the main differences between the cells of the preimplantation embryo and cells from later divisions is the organization of the mitotic spindle. The mouse mitotic spindle is acentrosomal and self organises into a barrel-shaped structure [28], [29]. Spindle microtubules polymerise from multiple microtubule organising centres. This unique spindle structure is present during the first three post-zygotic cell divisions where the Wt Y chromosome is most unstable [14]. One possible hypothesis is that there are fewer spindle microtubules that attach to the kinetochores, creating an impaired centromere that is susceptible to chromosome segregation errors. The length of the mitotic spindle may also be a contributing factor. The first four cell divisions have an elongated spindle length of around 25 µm compared with 10 µm for the blastocyst stage cells [29]. Furthermore, there may also be a limited pool of kinetochore proteins because of the transition from maternal to embryonic gene expression [30]. Once the embryonic cells transition to a centrosomal spindle formation then the impaired centromere functions normally.

It also appears that a large reduction in centromeric alpha satellite DNA can be tolerated in human individuals. A population screening study of several thousand centromeres showed that the frequency of low levels of alpha satellite DNA was unusually common in chromosome 21 [31]. Functional centromere proteins, CENP-C and CENP-E were present at chromosome 21 centromeres with reduced alpha satellite, but a quantification analysis was not performed. It is unclear whether this finding has any functional significance regarding the high rates of chromosome 21 non-disjunction during maternal meiosis I.

What happens when too much centromere DNA is deleted? Several examples in the literature suggest that a partial deletion of alpha satellite DNA in human cytogenetic cases may have triggered a shift in centromere position to form a pseudo-neodicentric chromosome [32], [33], [34]. So it appears that there may be a lower threshold for centromere stability, which then renders the centromere completely inactive. However, we cannot say conclusively that the partially deleted centromere DNA in these cases has caused the centromere shift because the progenitor centromere DNA has not been identified and analysed. In contrast, the Wt mouse has provided a spontaneous mutant where the progenitor Y chromosome is available for characterisation. Experimental serial deletions of a native mammalian centromere provides another possible method to functionally dissect the centromere, but remains challenging due to the repeat rich nature of the centromere DNA. These experiments may be possible in cell lines carrying human neocentromeres, or in natural centromeres with a low repeat DNA density such as in the chicken chromosomes, 5, 27 and Z [35]. Fukagawa and colleagues have utilised the DT40 cell line system which allows easy manipulation of the DNA to conditionally delete an entire centromere [36]. Smaller deletions of the centromere may assist in identifying the minimal amount of centromere DNA required for stable chromosome segregation. However, the information gained from these experiments would be limited to individual model systems since centromere size and CENP-A amount differs between species.

In humans, the chromosome Y alpha satellite array and CENP-A sizes are markedly reduced when compared to other chromosomes [37], [38]. This smaller array length does not appear to have any significant affect on chromosome missegregation rates apart from aging somatic tissues [39], [40], [41]. Y centromere array size and Y disomy has also been examined but no correlation has been observed for both 47,XYY individuals and sperm Y disomy rates in normal individuals [42]. A limitation with this latter study is that extreme reduction of Y alpha satellite was not examined, nor was the fathers’ Y centromere array in the 47,XYY cases. It would be interesting to test whether *de novo* Y centromere deletions appear in individuals with Y chromosome instability.
